# The Antidiabetic and Antinephritic Activities of *Tuber melanosporum* via Modulation of Nrf2-Mediated Oxidative Stress in the db/db Mouse

**DOI:** 10.1155/2018/7453865

**Published:** 2018-07-29

**Authors:** Xue Jiang, Shanshan Teng, Xue Wang, Shan Li, Yaqin Zhang, Di Wang

**Affiliations:** School of Life Sciences, Jilin University, Changchun 130012, China

## Abstract

*Tuber melanosporum* (TM), a valuable edible fungus, contains 19 types of fatty acid, 17 types of amino acid, 6 vitamins, and 7 minerals. The antidiabetic and antinephritic effects of TM and the underlying mechanisms related to oxidative stress were investigated in db/db mice. Eight-week oral administration of metformin (Met) at 0.1 g/kg and TM at doses of 0.2 and 0.4 g/kg decreased body weight, plasma glucose, serum levels of glycated hemoglobin, triglyceride, and total cholesterol and increased serum levels of high-density lipoprotein cholesterol in the mice, suggesting hypoglycemic and hypolipidemic effects. TM promoted glucose metabolism by increasing the levels of pyruvate kinase and hepatic glycogen. It also regulated the levels of inflammatory factors and oxidative enzymes in serum and/or the kidneys of the mice. Additionally, TM increased the expression of nuclear respiratory factor 2 (Nrf2), catalase, heme oxygenase 1, heme oxygenase 2, and manganese superoxide dismutase 2 and decreased the expression of protein kinase C alpha, phosphor-janus kinase 2, phosphor-signal transducer and activator of transcription 3, and phosphor-nuclear factor-*κ*B in the kidneys. The results of this study reveal the antidiabetic and antidiabetic nephritic properties of TM via modulating oxidative stress and inflammation-related cytokines through improving the Nrf2 signaling pathway.

## 1. Introduction

The incidence of diabetes mellitus (DM), a metabolic disturbance disease characterized by chronic hyperglycemia [1], has increased rapidly worldwide. Currently, a global population of 382 million people are diagnosed with DM and this number is predicted to rise to 592 million by 2035 [2]. Type 2 diabetes mellitus (T2DM), which is associated with *β*-cell dysfunction and insulin resistance and/or insulin secretion deficiency, is the most common form of DM [3]. Prolonged hyperglycemia in DM results in various secondary complications including nephropathy, hepatic damage, retinopathy, neuropathy, and cardiovascular disease [4–6]. Diabetic nephropathy (DN), a leading cause of end-stage renal disease, is the most common diabetic microvascular complication with high mortality and morbidity [7]. The progression of DN is associated with hyperglycemia, hyperlipidemia, and oxidative stress [8]. Renal inflammation resulting from the accumulation of inflammatory cells in the kidney has been reported as a key factor in the development of DN [9]. Furthermore, mesangial expansion and renal tubule damage, the major morphological alterations of DN, are associated with oxidative stress [10]. Hyperglycemia leads to the overproduction of mitochondrial superoxide, which causes the accumulation of intracellular reactive oxygen species (ROS) that are responsible for the defective angiogenesis and inflammatory pathway activation [11].

Current antidiabetic therapies have some limitations. Moreover, DM is a chronic disease with miscellaneous complications that require long-term treatment. Some effective Western medicines for diabetes are associated with high cost and adverse effects [12]. Furthermore, many treatments, such as oral antihyperglycemic agents and insulin injections, only address blood glucose regulation and *β*-cell function and have little therapeutic effect on complications [13]. Therefore, it is necessary to find alternative agents for the treatment of diabetes and its complications that have lower costs and fewer side effects.

Edible fungi have been used as folk tonic foods and/or medicines to prevent and/or treat diseases due to their efficacy and auxiliary therapeutic effects with few adverse effects [14, 15]. *Tuber melanosporum* (TM), an edible fungus containing many nutritional components [16], has been shown to exhibit antiviral, antimutagenic, antimicrobial, and anti-inflammatory activities [17]. However, the antidiabetic and antinephritic activities of TM and their underlying mechanisms have not yet been reported.

In this study, leptin receptor deficient (db/db) mice, which are systemic mutation mice that develop hyperinsulinemia and insulin resistance at 2 weeks old and then exhibit *β*-cell failure and hyperglycemia after 4 weeks [18], were used as a model to observe the effects of TM on diabetes and diabetic nephropathy and expose underlying mechanisms related to oxidative stress. Our results provide the first experimental evidence to support the development of TM as functional food for adjuvant therapy for diabetes and diabetic nephropathy.

## 2. Materials and Methods

### 2.1. Measurement of the TM Components

A TM fruiting body (purchased from Senzhong Co. Ltd., Yunnan, China) was pulverized in a crushing machine and stored in a dryer for the subsequent experiment.

#### 2.1.1. Main Component Analysis

The quantities of the main TM components including total sugar, reducing sugar, protein, total ash, crude fat, crude fiber, and total polyphenols were measured using the phenol-sulfuric acid method [19], direct titration [20], the Kjeldahl method [21], combustion [22], Soxhlet extraction [23], double differences method [24], and the Folin-Ciocalteu method [25], respectively. The quantities of triterpenoids, mannitol, and vitamins were measured using high-performance liquid chromatography (HPLC) [26–28]. Total flavonoids, carotenoids, and sterols were measured using UV spectrophotometry [29–31].

#### 2.1.2. Fatty Acid Analysis

TM was extracted via reflux extraction at 80°C with 2% NaOH in a methanol solution, and then a 14% BF3 solution was added and the mixture was incubated for another 10 min. After cooling to room temperature, a saturated solution of NaCl and n-heptane was added. The collected supernatant was mixed with anhydrous sodium sulfate, and the levels of fatty acids were analyzed using gas chromatography (GC, Agilent 7890A) [32].

#### 2.1.3. Amino Acid Analysis

TM was hydrolyzed using 6 mol/L of HCl at 110°C for 24 h. After vacuum drying, the samples were dissolved in 1 mL hydrochloric acid (HCl) solution (0.02 mol/L), which was mixed with triethylamine acetonitrile (1 mol/L) and phenyl isothiocyanate (0.1 mol/L) in a ratio of 2 : 1 : 1 (V : V : V). After the addition of 2 mL n-hexane for 10 min, a quantitative analysis of the amino acids was carried out by HPLC (Agilent 1260; column: Agilent C18 (4.6 × 250 mm × 5 *μ*m); mobile phase A: 0.1 mol/L sodium acetate solution/acetonitrile (1 : 1); mobile phase B: acetonitrile/ultrapure water (8 : 2); flow rate: 1.0 mL/min) [33].

#### 2.1.4. Mineral Analysis

After pretreating the TM with hydrogen nitrate for 27 min (3 min at 100°C, 3 min at 140°C, 3 min at 160°C, 3 min at 180°C, and 15 min at 190°C), the levels of zinc, iron, manganese, calcium, copper, sodium, and potassium were detected using inductively coupled plasma optical emission spectrometry (ICP-OES, Optima 8000) [34], and lead, mercury, chromium, arsenic, cadmium, and selenium were analyzed using inductively coupled plasma mass spectrometry (ICP-MS, Thermo Fisher Scientific ICAPQ) [35].

### 2.2. Animal Care and Experimental Design

The experimental animal protocol was approved by the Ethical Committee of Animal Research of Jilin University (20170301). All efforts were made to minimize animal suffering and reduce the number of animals used, according to the recommendations of Laboratory Animal Care and Use. The db/db mice and wild db/+ littermates (8 weeks, male, SCXK (Su) 2015-0001) were purchased from the Nanjing Biomedical Research Institute of Nanjing University, Nanjing, China. The db/db mice develop hyperinsulinemia and insulin resistance at 2 weeks age and then exhibit *β*-cell failure and hyperglycemia after 4 weeks. Therefore, the db/db mice were chosen to be a common diabetes model to accurately reflect the pathophysiology of diabetes [18]. All of the mice were housed on a 12-h light-dark cycle (lights on 07:00–19:00) and food available (growth and reproduction fodder of mice) ad libitum in a quiet room at a temperature of 23 ± 1°C and humidity of 60%.

**Figure 1:**
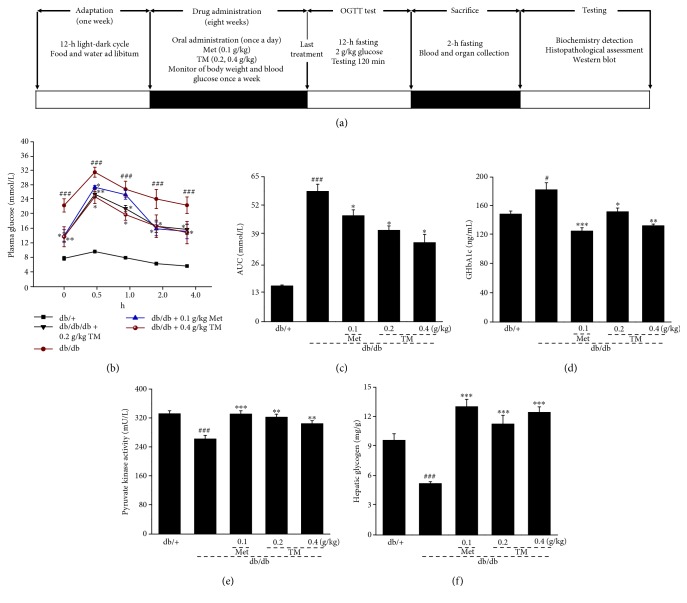
(a) The drug administration and study protocol. Eight-week TM treatment affected the (b) oral glucose tolerance, (c) AUC, (d) the serum levels of GHbA1c, (e) the serum levels of PK, and (f) the content of hepatic glycogen in db/db mice compared to db/+ mice. The data were analyzed using post hoc test of Holm-Sidak, and they are expressed as means ± SEMs (*n* = 10). ^#^*p* < 0.05 and ^###^*p* < 0.001 versus db/^+^ mice; ^∗^*p* < 0.05, ^∗∗^*p* < 0.01, and ^∗∗∗^*p* < 0.001 versus nontreated db/db mice. TM: *T. melanosporum*; AUC: the area under the curve of glucose of oral glucose tolerance; GHbA1c: glycosylated hemoglobin A1c; PK: pyruvate kinase.

The drug administration and study protocol are shown in Figure 1(a). Doses and the administration route were selected based on preliminary experiments performed in our laboratory. After one week adaptation, the db/+ mice were given physiological saline by oral administration for eight weeks and served as the control group (*n* = 12). The db/db mice with blood glucose levels > 11.1 mmol/L were randomly divided into four groups (*n* = 12/group) and orally administered with physiological saline (model group), Met at 0.1 g/kg (positive control group), and TM at doses of 0.2 and 0.4 g/kg once a day for eight weeks.

### 2.3. Oral Glucose Tolerance Test in db/db Mice

The oral glucose tolerance test (OGTT) was performed in overnight-fasted db/db mice after the last drug administration. All mice were orally administered with 2.0 g/kg of glucose. Blood samples were collected from the tail vein at 0, 30, 60, 120, and 240 min and assayed using a fast blood glucose meter [36]. The formula used for determining the area under the blood glucose curve (AUC) was as follows [37]: AUC = (basal glycemia + glycemia 0.5 h) × 0.25 + (glycemia 0.5 h + glycemia 1 h) × 0.25 + (glycemia 1 h + glycemia 2 h) × 0.5.

### 2.4. Sample Collection and Parameter Determination

Blood was sampled via the tail vein before the mice were sacrificed. After centrifugation of the blood samples at 3000 rpm for 10 min, the serum was collected and stored at −80°C for further use. After sacrifice, the kidney and liver tissues were collected and one part of the tissues was washed in ice-cold physiological saline solution and then homogenized in double-distilled water and/or a radioimmunoprecipitation assay buffer (RIPA; Sigma-Aldrich, USA) containing 1% protease inhibitor cocktail and 2% phenylmethanesulfonyl fluoride (Sigma-Aldrich, USA).

The levels of interleukins (ILs) IL-2 (cat. number CK-E20010), IL-6 (cat. number CK-E20012), and IL-10 (cat. number CK-E20005), 6-keto-prostaglandin F1*α* (6-K-PGF1*α*; cat. number CK-E30144), monocyte chemoattractant protein-5 (MCP-5; cat. number CK-E95264), matrix metalloproteinase-9 (MMP-9; cat. number CK-E90157), urine N-acetyl-*β*-D-glucosidase (NAG; cat. number CK-E20276), and ROS (cat. number CK-E91516) in the kidney; the levels of glycated hemoglobin A1c (GhbA1c; cat. number CK-E20512), triglyceride (TG; cat. number CK-E91733), total cholesterol (TC; cat. number CK-E91839), high-density lipoprotein cholesterol (HDL-C; cat. number CK-E93031), low-density lipoprotein cholesterol (LDL-C; cat. number CK-E93032), and pyruvate kinase (PK; cat. number CK-E20312) in serum; and the levels of superoxide dismutase (SOD; cat. number CK-E20348), glutathione peroxidase (GSH-Px; cat. number CK-E92669), and catalase (CAT; cat. number CK-E92636) in the kidney and serum were detected by enzyme-linked immunosorbent assay (ELISA) according to the manufacturer's instructions (Shanghai Yuanye Bio-Technology Co. Ltd., Shanghai, China). The concentration of hepatic glycogen (HG; cat. number A043) was detected by the procedures provided by the manufacturer of the assay kit (Nanjing Jiancheng Bioengineering Institute, Nanjing, China) [38].

### 2.5. Histopathological Observation of the Kidneys

Histologic assessment of the kidneys was carried out as in a previous study [39]. Briefly, tissues were fixed with 10% neutral phosphate-buffered formalin for 48 h, embedded in paraffin, and sliced into 5 *μ*m thick sections. After staining with hematoxylin and eosin (H&E) and periodic acid Schiff (PAS), histopathological examination was carried out using optical microscopy.

### 2.6. Western Blot

One part of the collected kidney tissue was homogenized in RIPA containing 1% protease inhibitor cocktail and 2% phenylmethanesulfonyl fluoride. Protein concentrations were determined by the Bradford method, and 40 *μ*g of protein was separated using 12% SDS-PAGE gel and electroblotted onto a nitrocellulose membrane (0.45 *μ*m; Bio Basic Inc., USA). After blocking with 5% BSA for 4 h, the transferred membranes were incubated with the following primary antibodies overnight at 4°C at a dilution of 1 : 2000: phosphor-janus kinase 2 (p-JAK2, ab68268), total-janus kinase 2 (t-JAK2, ab39636), phosphor-nuclear factor-*κ*B (p-NF-*κ*B, ab86299), total-NF-*κ*B (t-NF-*κ*B, ab32536), phosphor-signal transducer and activator of transcription 3 (p-STAT3, ab76315), total-STAT3 (t-STAT3, M06-596), catalase (CAT, ab16731), heme oxygenase 1 (HO-1, ab68477), nuclear respiratory factor 2 (Nrf2, ab137550), manganese superoxide dismutase 2 (SOD2, ab13533), heme oxygenase 2 (HO-2, ab90492), protein kinase C alpha (PKC-*α*, ab23513) (Abcam, Cambridge, USA), and glyceraldehyde-3-phosphate dehydrogenase (GAPDH; ABS16) (Merck Millipore, Darmstadt, Germany). After 5 washes with TBST buffer, the transferred membranes were incubated with horseradish peroxidase-conjugated goat anti-rabbit secondary antibody (sc-3836) (Santa Cruz Biotechnology, Santa Cruz, USA) for 4 h at 4°C. Chemiluminescence was detected using immobilon Western HRP substrate (Millipore Corporation, Billerica, USA). The intensity of the bands was quantified by scanning densitometry using an imaging system (Ultra-Violet Products Ltd., Cambridge, UK).

### 2.7. Statistical Analysis

Data were analyzed using SPSS 16.0 software (IBM Corporation, Armonk, USA), and continuous variables were expressed as mean ± SEM. A homoscedasticity test was carried out. A post hoc Holm-Sidak test was used to calculate statistical significance. A *p* value under 0.05 was considered statistically significant.

## 3. Results

### 3.1. Composition of TM

The TM contained 35.60% total sugars, 2.90% reducing sugar, 13.10% protein, 6.60% total ash, 7.30% crude fat, 5.00% crude fiber, 0.59% total flavones, 0.04% total triterpenoids, 1.25% mannitol, 0.67% total polyphenols, 3.20 × 10^−4^% carotenoids, and 3.58% total sterols (Table 1). Among the 35 types of fatty acid detected, only 19 were found in the TM sample (Table 2). Among 17 types of detected amino acid, glutamic acid, aspartic acid, and lysine were present at higher concentrations than others (Table 3). Among 9 detected vitamins, the three most common were vitamins C, B_3_, and D_2_ (Table 4). The minerals Zn, Fe, Mn, Ca, Cu, Na, and K were also detected (Table 5), and the concentrations of the heavy metals Pb, Hg, Cr, As, and Cd were lower than the detection limits of traditional analytical techniques, which indicates the safety of using TM (Table 5).

### 3.2. The Hypoglycemic Effect of TM on db/db Mice

Compared with the db/+ mice, the db/db mice had increased body weights and plasma glucose levels and reduced organ indexes of the spleen and kidney (*p* < 0.001, Table 6). After 8 weeks oral administration, 0.4 g/kg of TM reduced body weight by over 11% and plasma glucose by over 30% (*p* < 0.05, Table 6). The reduced indexes of the spleen and kidneys of the db/db mice were strongly improved by 0.4 g/kg of TM administration (*p* < 0.05, Table 6).

As a more sensitive indicator of early abnormalities of glucose regulation than fasting blood glucose, the OGGT was conducted after 8 weeks administration of TM to confirm its antihyperglycemic capacity [40]. The concentration of fasting blood glucose in the db/db mice was significantly higher than that of the db/+ mice within 4 h of 2.0 g/kg glucose administration. Similar to the Met group, the TM-treated db/db mice showed a significant reduction in blood glucose levels from 30 min to 4 h (*p* < 0.05, Figure 1(b)). Compared to the db/db mice, a significantly low AUC was noted in the TM and Met-treated mice (*p* < 0.05, Figure 1(c)). High serum levels of GHbA1c were observed in the db/db mice (*p* < 0.05, Figure 1(d)); 0.1 g/kg of Met and 0.2 g/kg and 0.4 g/kg of TM reduced GHbA1c levels by 31.2% (*p* < 0.001), 16.6% (*p* < 0.05), and 27.2% (*p* < 0.01), respectively (Figure 1(d)).

PK can promote carbohydrate metabolism by contributing to the glycolytic pathway [41]. Compared to the nontreated db/db mice, the mice that received 8 weeks TM administration exhibited a >16.1% increase in serum PK levels (*p* < 0.01, Figure 1(e)) and an increase in HG content of >120% (*p* < 0.001, Figure 1(f)).

### 3.3. The Hypolipidemic Effects of TM in db/db Mice

**Figure 2:**
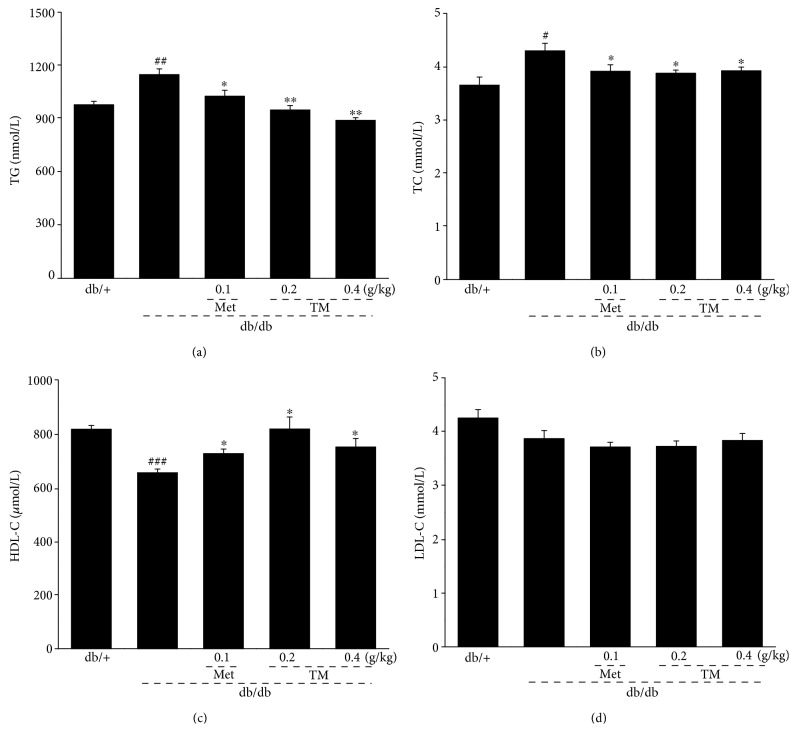
Eight-week TM treatment regulated the levels of the (a) TG, (b) TC, (c) HDL-C, and (d) LDL-C in the serum of db/db mice. The data were analyzed using post hoc test of Holm-Sidak, and they are expressed as means ± SEMs (*n* = 10). ^#^*p* < 0.05, ^##^*p* < 0.01, and ^###^*p* < 0.001 versus db/+ mice; ^∗^*p* < 0.05 and ^∗∗^*p* < 0.01 versus nontreated db/db mice. TM: *T. melanosporum*; TG: triglyceride; TC: total cholesterol; HDL-C: high-density lipoprotein cholesterol; LDL-C: low-density lipoprotein cholesterol.

As diabetes mellitus is commonly accompanied by hyperlipidemia [42], we determined the levels of serum TG, TC, HDL-C, and LDL-C to analyze the antihyperlipidemic activities of TM. Compared to the nontreated db/db mice, except for LDL-C, TM reduced the levels of TG (*p* < 0.01, Figure 2(a)) and TC (*p* < 0.05, Figure 2(b)) and enhanced the levels of HDL-C (*p* < 0.05, Figure 2(c)) in serum.

### 3.4. Renal Protective Effects of TM in db/db Mice

**Figure 3:**
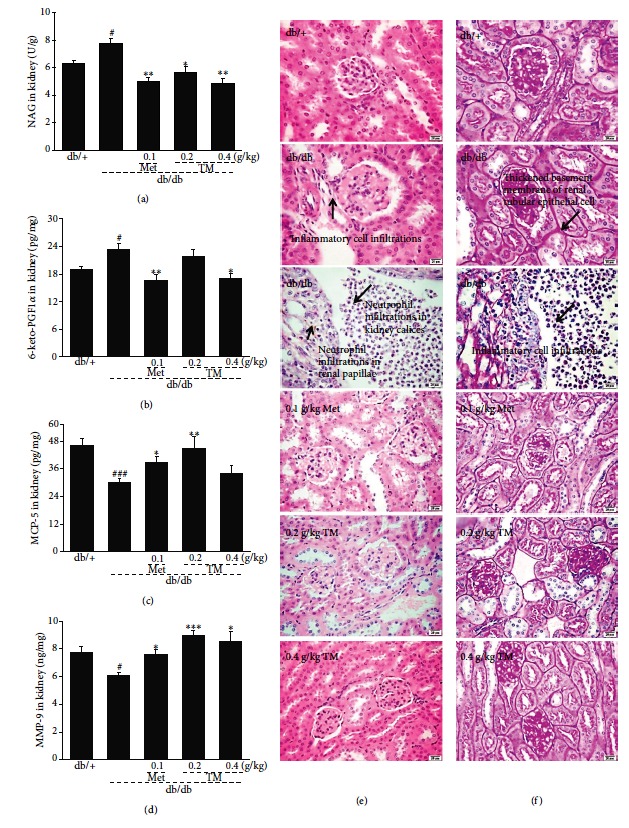
Eight-week TM treatment affected the levels of the (a) NAG, (b) 6-keto-PGF1*α*, (c) MCP-5, and (d) MMP-9 in the kidney of db/db mice. The data were analyzed using post hoc test of Holm-Sidak, and they are expressed as means ± SEMs (*n* = 10). ^#^*p* < 0.05 and ^###^*p* < 0.001 versus db/+ mice; ^∗^*p* < 0.05, ^∗∗^*p* < 0.01, and ^∗∗∗^*p* < 0.001 versus nontreated db/db mice. Histopathological analysis in kidney was shown by (e) H&E staining (scale bar: 20 *μ*m; magnification: 400x) and (f) PAS staining (scale bar: 20 *μ*m; magnification: 400x). TM: *T. melanosporum*; NAG: n-acetyl-*β*-d-glucosaminidase; 6-keto-PGF1*α*: 6-keto prostaglandin F1*α*; MCP-5: monocyte chemotactic protein-5; MMP-9: matrix metalloproteinase-9; H&E: Hematoxylin and eosin; PAS: periodic acid Schiff.

NAG is a lysosomal enzyme in proximal tubular cells and serves as an index of intense tubular damage in the early stages [43]. Similar to Met treatment, 0.4 g/kg of TM reduced the levels of NAG in the kidneys of the db/db mice by 37.6% (*p* < 0.01, Figure 3(a)). 6-keto-PGF1*α*, MCP-5, and MMP-9 can also be used as valuable indicators of renal injury in DN. The 0.4 g/kg of TM treatment caused a 27.6% reduction in serum levels of 6-keto-PGF1*α* in the db/db mice (*p* < 0.05, Figure 3(b)); 0.2 g/kg of TM treatment led to 50.6% and 47.6% increases in MCP-5 (*p* < 0.01, Figure 3(c)) and MMP-9 (*p* < 0.001, Figure 3(d)) serum levels in the db/db mice. Treatment with TM at 0.2 g/kg exhibited better effects on MCP-5 and MMP-9 levels in the kidney than did 0.4 g/kg (*p* < 0.01, Figures 3(c) and 3(d)). H&E and PAS staining of the kidney further confirmed the renal protective effect of TM. Many neutrophil infiltrations in the kidney calices and renal papillae were observed in the db/db mice, which significantly improved after TM and Met administration (Figure 3(e)). The PAS staining results showed that the thickened basement membrane of renal tubular epithelial cells and inflammatory cell infiltrations in the db/db mice were relieved by Met and TM (Figure 3(f)).

**Figure 4:**
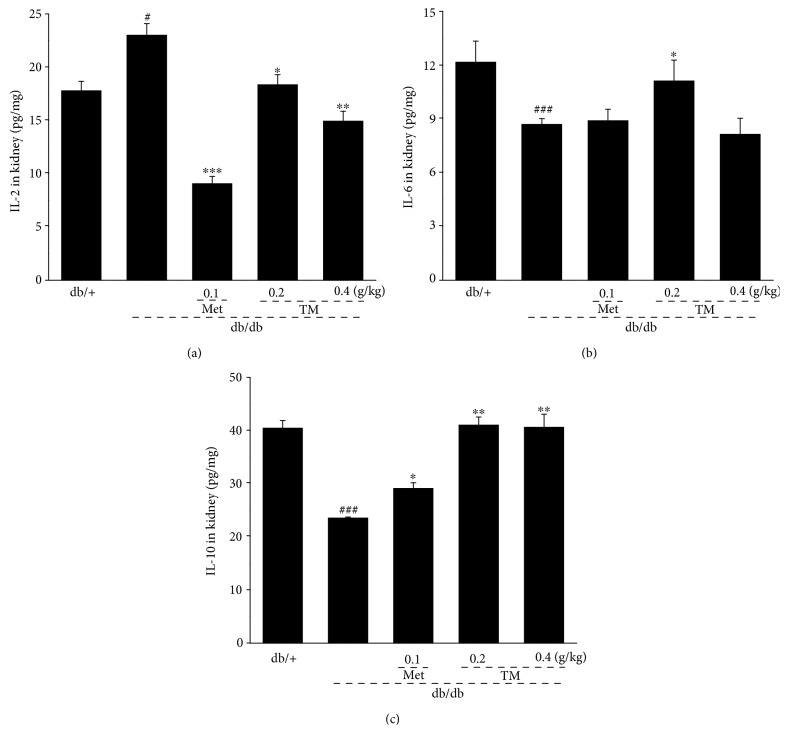
Eight-week TM treatment affected the levels of the (a) IL-2, (b) IL-6, and (c) IL-10 in the kidney of db/db mice. The data were analyzed using post hoc test of Holm-Sidak, and they are expressed as means ± SEMs (*n* = 10). ^#^*p* < 0.05 and ^###^*p* < 0.001 versus db/+ mice; ^∗^*p* < 0.05, ^∗∗^*p* < 0.01, and ^∗∗∗^*p* < 0.001 versus nontreated db/db mice. TM: *T. melanosporum*; IL-2: interleukin-2; IL-6: interleukin-6; IL-10: interleukin-10.

Typical hyperglycemia and hyperlipidemia of DN always result in glomerular injury associated with severe inflammation [44]. The release of inflammatory cytokines in the kidneys of the db/db mice was regulated by the 8-week TM treatment: 0.2 g/kg of TM strongly reduced the level of IL-2 by 20.2% (*p* < 0.05, Figure 4(a)) and increased the levels of IL-6 and IL-10 by 27.9% (*p* < 0.05, Figure 4(b)) and 74.4% (*p* < 0.01, Figure 4(c)), respectively. Only TM at 0.2 g/kg significantly improved IL-6 levels in the kidney.

### 3.5. The Antioxidative Effects of TM in the db/db Mice

Overproduction of ROS and hypoactivities of SOD, GSH-Px, and CAT were observed in the serum and/or kidneys of the db/db mice (*p* < 0.05, Table 7). TM, especially at 0.4 g/kg, increased serum levels of SOD and CAT by 12.9% and 17.5%, respectively (*p* < 0.001, Table 7); however, no significant influence on the serum levels of GSH-Px was noted in the TM-treated db/db mice. Additionally, TM significantly reduced the high levels of ROS in the kidney by 30.1% after 8 weeks administration (*p* < 0.05, Table 7).

### 3.6. Regulation of Nrf2 and NF-*κ*B Signaling by TM in db/db Mice

**Figure 5:**
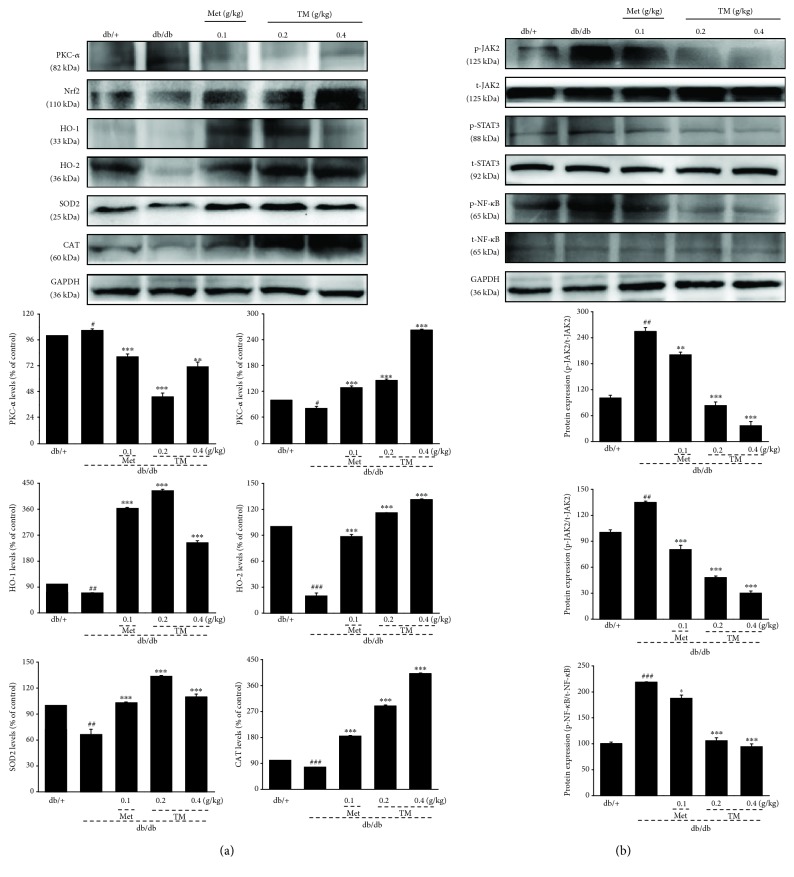
Eight-week TM treatment regulated the expressions of (a) CAT, HO-1, HO-2, SOD2, Nrf2, and PKC-*α* and (b) phosphor-JAK2, phosphor-NF-*κ*B, and phosphor-STAT3 in the kidney of db/db mice. The data on quantified protein expressions were normalized to the levels of GAPDH and related total proteins. The data were analyzed using post hoc test of Holm-Sidak, and they are expressed as means ± SEMs (*n* = 10). ^#^*p* < 0.05, ^##^*p* < 0.01, and ^###^*p* < 0.001 versus db/+ mice; ^∗^*p* < 0.05, ^∗∗^*p* < 0.01, and ^∗∗∗^*p* < 0.001 versus nontreated db/db mice. TM: *T. melanosporum*; CAT: catalase; HO-1: heme oxygenase 1; HO-2: heme oxygenase 2; SOD2: superoxide dismutase 2; Nrf2: nuclear respiratory factor 2; PKC-*α*: protein kinase C alpha; JAK2: janus kinase 2; NF-*κ*B: nuclear factor-*κ*B; STAT3: signal transducers and activators of transcription 3.

To understand the underlying mechanisms of the antidiabetic and antidiabetic nephritic effects of TM, the expression levels of proteins related to Nrf2 and NF-*κ*B signaling in the kidney were assessed by Western blotting. Compared with the db/db mice, TM visibly upregulated the expressions of CAT, HO-1, HO-2, SOD2, and Nrf2 (*p* < 0.001, Figure 5(a)) and downregulated the expressions of PKC-*α* (*p* < 0.01, Figure 5(a)), p-JAK2, p-STAT3, and p-NF-*κ*B in the kidneys (*p* < 0.001, Figure 5(b)).

## 4. Discussion

In the present study, we first confirmed the antidiabetic and antidiabetic nephritic effects of TM in db/db mice and clarified the underlying mechanisms associated with oxidative stress. Compared with currently used effective medicines (such as Met), TM contains various nutritional ingredients including 19 types of fatty acid, 17 types of amino acid, 6 vitamins, and 7 minerals. Its nature as a crude agent suggests that it has multieffective components, which might target many molecules in the signaling of inflammation and oxidative stress. This “systemic targeting” will eliminate the inflammation and oxidative stress in a much more “natural” way, so fewer adverse side effects are expected. The use of TM as a folk tonic food has been practiced by Europeans for thousands of years, which further emphasizes its safety and lack of adverse side effects. The “systemic targeting” may also explain the nondose-dependent manner by which TM displayed its antidiabetic and antidiabetic nephritic effects. A number of natural products are reported to show various pharmacological activities in a nondose-dependent manner [45, 46].

TM effectively decreased the body weights and food intakes of the db/db mice. The hypoglycemic activity of TM was confirmed by the reduction in blood glucose and the modulation of the OGTT and GHbA1c levels. TM enhanced the levels of HG and PK in serum. PK promotes carbohydrate metabolism via its contribution to the glycolytic pathway and is a key glycolytic enzyme in glucose homeostasis [47]. As glycogen is the main intracellular storable form of glucose, the induction of glycogen accumulation in hepatocytes is an important antihyperglycemic phenomenon [38]. Furthermore, as a popular type 2 diabetic model, db/db mice develop insulin resistance, which leads to hyperglycemia and hyperinsulinemia [48]. Although the etiologies of DN are complex, dyslipidemia, characterized by abnormal lipid profiles, has been reported as a crucial factor in kidney damage [49, 50]. The characteristic features of diabetic lipid profiles are high levels of TG, TC, or LDL-C in serum and tissues [51]. Eight-week TM administration exhibited strongly hypolipidemic effects in db/db mice, suggesting renal protection.

Our data show that TM exerted strong renal protection by regulating the levels of NAG, 6-keto-PGF1*α*, MCP-5, and MMP-9. NAG and 6-keto-PGF1*α* serve as specific and sensitive indicators of the extent of oxidative damage and acute kidney damage [52, 53]. The accumulation of extracellular matrix is a pathological characteristic of DN. MMP-9 protects mice by promoting the decomposition of glomeruli fibrin caps during glomerulonephritis via fibrinolytic activity [54]. MCP-5 is reported to be a murine homolog of human MCP-1, which is secreted by renal tubular epithelial cells and kidney mesangial cells during the process of inflammation and is differentially expressed in the kidneys [55]. Cytokines, as small proteins with multipotent biological features, including interleukins and interferons, show anti-inflammatory or proinflammatory properties [56]. Interleukins play an important role during the development of inflammation. The overproduction of IL-2 activates proinflammatory CD4+, which exacerbates glomerular damage via recruiting macrophages and neutrophils [57]. IL-6 can enhance basic and insulin-stimulated glucose uptake and has favorable effects on energy metabolism [58]. As an inhibitor of TNF-*α* expression, IL-10 is recognized as an efficient anti-inflammatory cytokine, which can ameliorate insulin resistance and hyperglycemia [59, 60]. Eight-week TM administration successfully regulated the levels of interleukins in the kidneys of db/db mice, further confirming its renal protective effects. In diabetes, hyperglycemia activates protein kinase C (PKC), which increases the expression of NF-*κ*B and induces both cytokines and chemokines [61], which contribute to the accumulation of the extracellular matrix and injury of podocytes in diabetic animals [62]. The JAK/STAT system mediates abnormal kidney diseases, and suppression of JAK2 expression can relieve DN progression [63]. Phosphorylated JAKs result in the activation of full STAT activities [63]. It has been reported that NF-*κ*B activates STAT3 on tyrosine residues indirectly [64]. Our data suggest that TM-mediated renal protection in db/db mice may be related to its modulation of NF-*κ*B activation.

Oxidative stress, which is related to the overproduction of ROS, has been identified as a general pathogenic factor in DN [62, 65]. In contrast, antioxidant enzymes including SOD, GSH-Px, and CAT scavenge free radicals and prevent oxidative injury [66]. TM exhibited significant antioxidative effects in db/db mice by reducing ROS levels and increasing SOD, CAT, and GSH-Px levels in the kidneys and/or serum. Nrf2 is an important inducible transcription factor that protects redox homeostasis from oxidative injury [67]. Nrf2 combines with antioxidant response element (ARE) and mediates the expression of downstream genes HO-1 and SOD-1, which encode detoxifying and antioxidant enzymes in consort with relevant proteins [68, 69]. Subsequently, the overproduced ROS is scavenged via the activation of Nrf2 [70]. The inactivation of Nrf2 signaling is reported to be beneficial for the overactivation of NF-*κ*B [71].

In summary, we successfully explored the antidiabetic and antidiabetic nephritic properties of TM in db/db diabetic mice and found that these effects may be related to the modulation of oxidative stress and inflammation-related cytokines via Nrf2 signaling. The effects of TM are similar to those of Met, a commonly used hyperglycemic drug, which supports TM as a candidate nutritious natural product for DN adjunctive therapy.

## Figures and Tables

**Table 1:** Main components of TM.

Compounds	Contents (%)
Total sugar	35.60
Reducing sugar	2.90
Protein	13.10
Total ash	6.60
Crude fat	7.30
Crude fiber	5.00
Total flavones	0.59
Total triterpenoids	0.04
Mannitol	1.25
Total polyphenol	0.67
Carotenoid (×10^−4^)	3.20
Total sterol	3.58

TM: *T. melanosporum.*

**Table 2:** The compositions and percentage content of fatty acids in TM.

Compounds	Contents (%)	Compounds	Contents (%)	Compounds	Contents (%)
Octoic acid (C8:0)	ND^I^	Heptadecenoic acid (C17:1) (×10^−2^)	0.20	Docosanoic acid (C22:0) (×10^−3^)	0.02
Capric acid (C10:0)	ND^II^	Stearic acid (C18:0)	0.46	Eicosatrienoic acid (C20:3n6)	ND^X^
Undecanoic acid (C11:0)	ND^III^	*trans*-Oleic acid (C18:1n9t) (×10^−2^)	0.20	Erucic acid (C22:1n9) (×10^−2^)	0.30
Lauric acid (C12:0)	ND^IV^	Oleic acid (C18:1n9c)	1.79	Eicosatrienoic acid (C20:3n3)	ND^XI^
Tridecanoic acide (C13:0)	ND^V^	*trans*-Linoleic acid (C18:2n6t)	ND^VII^	Arachidonic acid (C20:4n6)	ND^XII^
Myristic acid (C14:0) (×10^−2^)	0.40	Linoleic acid (C18:2n6c)	3.85	Tricosanoic acid (C23:0) (×10^−2^)	0.40
Myristoleic acid (C14:1)	ND^VI^	Arachidic acid (C20:0)	0.05	Docosadienoic acid (C22:2n6)	ND^XIII^
Pentadecanoic acid (C15:0) (×10^−2^)	0.10	*γ*-Linolenic acid (C18:3n6)	ND^VIII^	Eicosapentaenoic acid (C20:5n3)	ND^XIV^
Pentadecenoic acid (C15:1)	ND^VII^	Eicosaenoic acid (C20:1n9)	0.03	Tetracosanoic acid (C24:0)	0.02
Hexadecanoic acid (C16:0)	0.64	*α*-Linolenic acid (C18:3n3)	ND^IX^	Nervonic acid (C24:1n9)	0.01
Palmitoleic acid (C16:1)	0.01	Heneicosanoic acid (C21:0) (×10^−2^)	0.30	Docosahexaenoic acid (C22:6n3)	ND^XV^
Heptadecanoic acide (C17:0) (×10^−2^)	0.70	Eicosadienoic acid (C20:2)	0.04		

ND: not detected; ND^I^: the detection limit was 4.20 mg/kg; ND^II^: the detection limit was 3.83 mg/kg; ND^III^: the detection limit was 3.54 mg/kg; ND^IV^: the detection limit was 2.99 mg/kg; ND^V^: the detection limit was 2.91 mg/kg; ND^VI^: the detection limit was 2.82 mg/kg; ND^VII^: the detection limit was 2.64 mg/kg; ND^VIII^: the detection limit was 2.51 mg/kg; ND^IX^: the detection limit was 2.36 mg/kg; ND^X^: the detection limit was 2.68 mg/kg; ND^XI^: the detection limit was 3.21 mg/kg; ND^XII^: the detection limit was 4.66 mg/kg; ND^XIII^: the detection limit was 2.88 mg/kg; ND^XIV^: the detection limit was 3.31 mg/kg; ND^XV^: the detection limit was 4.33 mg/kg.

**Table 3:** The compositions and percentage content of amino acids in TM.

Compounds	Contents (‰)	Compounds	Contents (‰)
Aspartic acid (Asp)	12.69	Proline (Pro)	5.77
Glutamic acid (Glu)	20.46	Tyrosine (Tyr)	5.53
Cystine (Cys)	5.75	Valine (Val)	5.76
Serine (Ser)	6.45	DL-methionine (Met)	1.61
Glycine (Gly)	7.48	Isoleucine (Ile)	4.38
Histidine (His)	3.69	Leucine (Leu)	6.48
Arginine (Arg)	9.94	Phenylalanine (Phe)	4.16
L-Threonine (Thr)	5.95	Lysine (Lys)	10.48
Alanine (Ala)	7.36		

TM: *T. melanosporum*.

**Table 4:** The compositions and percentage content of vitamins in TM.

Compounds	Contents (mg/kg)	Compounds	Contents (mg/kg)
Vitamin A	0.07	Vitamin B_1_	70.43
Vitamin B_2_	24.62	Vitamin B_3_	1533.01
Vitamin B_6_	ND^XVI^	Vitamin C	1706.52
Vitamin D_2_	196.64	Vitamin D_3_	ND^XVIII^
Vitamin E	ND^XVII^		

TM: *T. melanosporum*. ND^XVI^: the detection limit was 2.92 mg/kg; ND^XVII^: the detection limit was 1.32 mg/kg; ND^XVIII^: the detection limit was 0.084 mg/kg.

**Table 5:** The compositions and percentage content of minerals (including heavy metals) in TM.

Compounds	Contents (‱)	Compounds	Contents (*μ*g/kg)
Zinc (Zn)	1.04	Lead (Pb)	119.70
Iron (Fe)	1.03	Mercury (Hg)	219.62
Manganese (Mn)	0.08	Chromium (Cr)	5595.99
Calcium (Ca)	9.67	Arsenic (As)	75.06
Cupper (Cu)	0.68	Cadmium (Cd)	1417.40
Sodium (Na)	0.65	Selenium (Se)	ND^XIX^
Potassium (K)	208.10		

TM: *T. melanosporum*. ND^XIX^: not detected (the detection limit was 20 *μ*g/kg).

**Table 6:** Effects of 8-week TM treatment on the bodyweight, plasma glucose, and organ indices of mice.

	Week	db/+	db/db	0.1 g/kg Met	0.2 g/kg TM	0.4 g/kg TM
Body weights (g)	1	20.2 ± 0.4	43.2 ± 0.4^###^	42.9 ± 0.8	43.3 ± 0.8	43.6 ± 0.7
	3	21.4 ± 0.4	45.0 ± 0.7^###^	44.8 ± 0.7	46.5 ± 0.8	44.8 ± 0.7
	5	20.9 ± 0.4	48.0 ± 1.1^###^	43.9 ± 0.9^∗^	45.3 ± 1.2	43.6 ± 0.8^∗^
	7	21.7 ± 0.3	51.7 ± 1.0^###^	47.5 ± 1.2^∗^	50.2 ± 1.3	47.6 ± 0.8^∗^
	9	21.1 ± 0.7	55.7 ± 0.9^###^	52.9 ± 0.4^∗^	52.8 ± 1.4	49.5 ± 1.2^∗∗^

Plasma glucose (mmol/L)	1	5.6 ± 0.4	19.1 ± 1.4^###^	19.6 ± 1.2	17.4 ± 0.9	18.8 ± 2.1
	3	6.9 ± 0.4	19.6 ± 1.5^###^	18.8 ± 1.8	17.2 ± 0.9	18.8 ± 2.3
	5	6.5 ± 0.3	18.8 ± 1.2^###^	16.6 ± 1.1	15.8 ± 1.0	16.0 ± 1.5
	7	6.6 ± 0.4	21.7 ± 1.2^###^	16.8 ± 1.2^∗^	12.9 ± 1.2^∗∗^	15.1 ± 1.6^∗^
	9	6.5 ± 0.5	21.3 ± 1.1^###^	13.8 ± 1.5^∗^	14.0 ± 1.2^∗^	13.7 ± 1.7^∗^

Organ indexes (%)	Spleen	0.32 ± 0.06	0.11 ± 0.01^###^	0.13 ± 0.01^∗∗^	0.15 ± 0.02^∗∗^	0.17 ± 0.02^∗∗∗^
	Kidney	1.27 ± 0.07	0.70 ± 0.07^###^	0.79 ± 0.08^∗^	0.73 ± 0.07	0.84 ± 0.13^∗^

The data were analyzed using a one-way ANOVA and they are expressed as means ± SEMs (*n* = 10). ^###^*p* < 0.001 versus db/+ mice; ^∗^*p* < 0.05, ^∗∗^*p* < 0.01, and ^∗∗∗^*p* < 0.001 versus nontreated db/db mice. TM: *T. melanosporum.*

**Table 7:** The effects of *Tuber melanosporum* powder on oxidative stress-related factors in the serum and kidney of mice.

		db/+	db/db	0.1 g/kg Met	0.2 g/kg TM	0.4 g/kg TM
Serum	SOD (U/mL)	240.3 ± 5.0	193.6 ± 2.5^###^	245.7 ± 3.9^∗∗∗^	206.1 ± 3.9^∗^	218.6 ± 5.5^∗∗∗^
	GSH-Px (U/mL)	304.4 ± 9.5	230.8 ± 3.7^###^	227.2 ± 6.0	248.8 ± 11.6	236.5 ± 5.3
	CAT (U/mL)	54.6 ± 1.3	41.2 ± 0.9^###^	45.2 ± 1. 5^∗^	48.3 ± 1.9^∗∗^	48.4 ± 1.1^∗∗∗^

Kidney	ROS (U/mg)	46.1 ± 1.7	62.6 ± 4.1^#^	42.1 ± 2.1^∗∗∗^	51.1 ± 0.5	43.3 ± 3.2^∗^
	SOD (U/mg)	34.9 ± 2.8	23.3 ± 1.3^##^	29.4 ± 2.6^∗^	37.0 ± 5.0^∗^	34.1 ± 4.8^∗^
	GSH-Px (mg/mL)	75.7 ± 3.9	42.3 ± 1.3^###^	50.8 ± 3.0^∗^	71.6 ± 6.9^∗∗^	67.7 ± 6.4^∗∗^
	CAT (U/mg)	8.7 ± 0.5	7.0 ± 0.3^#^	8.8 ± 0.5^∗^	8.9 ± 0.4^∗∗^	9.0 ± 0.3^∗∗^

The data were analyzed using a one-way ANOVA and they are expressed as means ± SEMs (*n* = 10). ^#^*p* < 0.05, ^##^*p* < 0.01, and ^###^*p* < 0.001 versus db/+ mice; ^∗^*p* < 0.05, ^∗∗^*p* < 0.01, and ^∗∗∗^*p* < 0.001 versus nontreated db/db mice. TM: *T. melanosporum.*

## Data Availability

The data used to support the findings of this study are available from the corresponding author upon request.
